# Comparative Evaluation of Different Sanitizers Against *Listeria monocytogenes* Biofilms on Major Food-Contact Surfaces

**DOI:** 10.3389/fmicb.2019.02462

**Published:** 2019-11-07

**Authors:** Zi Hua, Ahmed Mahmoud Korany, Saadia Helmy El-Shinawy, Mei-Jun Zhu

**Affiliations:** ^1^School of Food Science, Washington State University, Pullman, WA, United States; ^2^Food Hygiene and Control Department, Faculty of Veterinary Medicine, Beni-Suef University, Beni Suef, Egypt

**Keywords:** biofilm, *L. monocytogenes*, sanitizers, food-contact surfaces, organic matter, peroxyacetic acid

## Abstract

Contaminated food-contact surfaces are recognized as the primary reason for recent *L. monocytogenes* outbreaks in caramel apples and cantaloupes, highlighting the significance of cleaning and sanitizing food-contact surfaces to ensure microbial safety of fresh produce. This study evaluated efficacies of four commonly used chemical sanitizers at practical concentrations against *L. monocytogenes* biofilms on major food-contact surfaces including stainless steel, low-density polyethylene (LDPE), polyvinyl chloride (PVC), polyester (PET), and rubber. In general, efficacies against *L. monocytogenes* biofilms were enhanced by increasing concentrations of quaternary ammonium compound (QAC), chlorine, and chlorine dioxide, or extending treating time from 1 to 5 min. The 5-min treatments of 400 ppm QAC, 5.0 ppm chlorine dioxide, and 200 ppm chlorine reduced 3.0–3.7, 2.4–2.7, and 2.6–3.8 log_10_ CFU/coupon *L. monocytogenes* biofilms depending on surfaces. Peroxyacetic acid (PAA) at 160 and 200 ppm showed similar antimicrobial efficacies against biofilms either at 1- or 5-min contact. The 5-min treatment of 200 ppm PAA caused 4.0–4.5 log_10_ CFU/coupon reduction of *L. monocytogenes* biofilms on tested surfaces. Surface material had more impact on the efficacies of QAC and chlorine, less influence on those of PAA and chlorine dioxide, while organic matter soiling impaired sanitizer efficacies against *L. monocytogenes* biofilms independent of food-contact surfaces. Data from this study provide practical guidance for effective disinfection of food-contact surfaces in food processing/packing facilities.

## Introduction

As a critical foodborne pathogen, *Listeria monocytogenes* causes approximately 1,600 cases of infection and 260 cases of death annually in the United States (Scallan et al., 2011). It has been implicated in multi-state outbreaks on fresh produce including cantaloupes (CDC, 2012), prepackaged caramel apples (CDC, 2015a), bean sprouts (CDC, 2015b), frozen vegetables (CDC, 2016a), and packaged salads (CDC, 2016b) since 2011. Contaminated food-contact surfaces, packing lines, and environment are incriminated as the primary reasons linked to *L. monocytogenes* outbreaks in fresh produce (McCollum et al., 2013; Angelo et al., 2017). Therefore, it is vital to sanitize food-contact surfaces along produce production lines effectively to ensure microbial safety of fresh produce.

Stainless steel (SS) and plastics are preferably used in the fresh produce industry due to their anti-fouling ability (FDA, 2008). SS, a corrosion-resistant metal, is an excellent material for food processing/packing equipment and extensively used in food industries such as fresh apple packing facilities (Jellesen et al., 2006). A conveyor belt, one of the most prevalent food-contact surfaces, directly contacts fresh produce and transports it to further processing or packing during post-harvest handling. Polyvinyl chloride (PVC), low-density polyethylene (LDPE), and rubber are FDA-approved food-contact substances that are extensively used as important components of conveyor belts (FDA, 2017). The conveyor belts around the optical sorting lines have been determined to be the major contamination sites in a minimally processed vegetable plant (Meireles et al., 2017). The brush bed, mostly made of polyester (PET), is an important and essential processing tool of the packing lines of fresh apples and other fruits. The contaminated brush-bed spray bar system was implicated in a recent caramel apple *L. monocytogenes* outbreak (Angelo et al., 2017). *L. monocytogenes* form biofilms on SS, PVC, LDPE, PET, and rubber surfaces (Krysinski et al., 1992; Beresford et al., 2001; Takahashi et al., 2010; Doijad et al., 2015; Papaioannou et al., 2018), exerting enhanced resistances to acid and sanitizer treatments (Ibusquiza et al., 2011; van der Veen and Abee, 2011), which makes routine disinfection in a food processing facility more difficult.

Food-contact surfaces are cleaned and disinfected daily with different chemical sanitizers in fresh produce processing plants and apple packing facilities. Peroxyacetic acid (PAA) is an environment-friendly sanitizer that decomposes and produces no harmful by-product (Dell’Erba et al., 2007). Quaternary ammonium compound (QAC) and chlorine are the most commonly used sanitizers for surface disinfections (Robbins et al., 2005; Olszewska et al., 2016; Dhowlaghar et al., 2018). Chlorine dioxide is considered as an alternative for chlorine due to its high oxidizing capacity (~2.5 times higher than that of chlorine) (Benarde et al., 1965). The bactericidal effects of the aforementioned sanitizers against *L. monocytogenes* biofilms on polystyrene surfaces were compromised in the presence of organic matter or when biofilm was aged (Korany et al., 2018). Different food-contact surfaces have unique physicochemical properties and hydrophobicity, which may provide unique harbor sites for *L. monocytogenes* during sanitizer intervention. Therefore, the objective of this study was to evaluate antimicrobial efficacies of four FDA-approved sanitizers against aged *L. monocytogenes* biofilms on major food-contact surfaces in the absence or presence of organic matter.

## Materials and Methods

### *L. monocytogenes* Strains and Cocktail Preparation

*Listeria monocytogenes* strain NRRL B-33069, NRRL B-57618, NRRL B-33006, NRRL B-33466, NRRL B-33071, and NRRL B-33385 were obtained from USDA-ARS culture collection of National Center (NRRL) for Agricultural Utilization Research (Peoria, IL, United States) and were stored at −80°C in Trypticase Soy Broth with 0.6% Yeast Extract (TSBYE, Fisher Scientific, Fair Lawn, NJ, United States) and 20% (v/v) glycerol. Each frozen culture was activated in TSBYE at 35 ± 2°C for 24 ± 2 h statically, then sub-cultured in TSBYE for additional 24 ± 2 h at 35 ± 2°C. The six-strain *L. monocytogenes* cocktail was prepared by mixing equal volumes of each activated strain, then centrifuged at 8,000 *× g* for 5 min at room temperature (22°C, RT). The resulting pellet was re-suspended in Modified Welshimer’s Broth (MWB, HiMedia, West Chester, PA, United States) to have a final population level of ~10^8^ CFU/ml.

### Surface Selection, Preparation, and Conditioning

The SS (AISI 316, No. 4 brushed finish) was obtained from the Washington State University Engineering Shops (Pullman, WA, United States). PVC, LDPE, and PET sheets were purchased from Interstate Plastics (Sacramento, CA, United States), and silicone rubber sheet was purchased from Rubber Sheet Warehouse (Los Angeles, CA, United States). All surface materials were cut into coupons of 15 mm × 7.5 mm at the Washington State University Engineering Shops.

To clean coupons, the prepared surface coupons were immersed in 100% methanol (Fisher Scientific) for 1 h, rinsed with sterile water three times, then immersed for 1 h in 70% ethanol (Fisher Scientific). The treated coupons were air dried under a biosafety cabinet overnight, which were ready for biofilm growth. To condition surface coupon with organic matter, the above cleaned surface coupons were immersed in 1:10 diluted apple juice or milk for 1 h at RT (Brown et al., 2014). After removing conditioning solution, coupons were air dried for 1 h at RT under a biosafety cabinet.

### *L. monocytogenes* Biofilm Formation

The above prepared coupons were subjected to a 15-min UV treatment in the biosafety hood to surface decontamination before inoculation with 2.0 ml of *L. monocytogenes* cocktail suspension in MWB (~10^8^ CFU/ml). The inoculated coupons in 24-well plates were incubated statically at RT for 7 days to grow *L. monocytogenes* biofilms without agitation (Abeysundara et al., 2018).

### Sanitizer Intervention Against *L. monocytogenes* Biofilms

Bioside HS (EnviroTech, Modesto, CA, United States) containing 15% PAA was used to prepare 160 and 200 ppm PAA solutions using sterile water. STOPIT (Pace International, Wapato, WA, United States) was diluted with sterile water to prepare 200 and 400 ppm QAC solutions. Chlorine solutions at 100 and 200 ppm were made from Accu-Tab (Pace International, Wapato, WA, United States), while 2.5 and 5.0 ppm chlorine dioxide solutions were generated on-site using chlorine dioxide generator donated by Pace International (Wapato, WA, United States). Concentration of PAA was verified using a AquaPhoenix Preacetic Acid test kit (Hanover, PA, United States), levels of QAC and chlorine were confirmed by the QAC and Chlorine test kits from LaMotte (Chestertown, MD, United States), and the concentration of chlorine dioxide solutions were measured by a HACH Chlorine Dioxide test kit (Loveland, CO, United States).

To evaluate the antimicrobial efficacy of sanitizers, 7-day-old *L. monocytogenes* biofilms on each surface coupon were washed with 2.0 ml of sterile phosphate buffered saline (PBS) three times and then immersed in 2.0 ml of each sanitizer solution for 1 or 5 min at RT. Coupons were first rinsed with 2.0 ml of Dey-Engley Neutralizing Broth (Oxoid, United States), then 2.0 ml sterile PBS immediately after sanitizer treatment. Four replicates were used for each surface material and sanitizer treatment, and triple independent experiments were conducted for each treatment combination.

### Biofilm Detachment and Enumeration

To detach and enumerate the *L. monocytogenes* cells in biofilm on the above treated coupons, the coupon in the respective well was transferred to 2-ml microtube containing 1.0 ml of sterile PBS and 3~4 glass beads. The tubes containing coupons were vigorously vortexed for 2 min using a benchtop mixer at the maximal speed. The detached bacterial suspension was 10-fold serially diluted with sterile PBS, and appropriate dilution was plated on TSAYE plates in duplicate. The plates were incubated at 35 ± 2°C for 48 h before enumeration.

### Statistical Analysis

Data were analyzed by uncorrected Fisher’s Least Significant Difference (LSD) to determine significant difference among groups at *p* ≤ 0.05 using Prism (Version 7.0, San Diego, CA, United States). Each experiment was repeated three times independently. Data were presented as an average from three independent studies and mean ± standard error mean (SEM) was reported.

## Results

### Efficacy of Quaternary Ammonium Compound Against *L. monocytogenes* Biofilms on Food-Contact Surfaces

**Figure 1:**
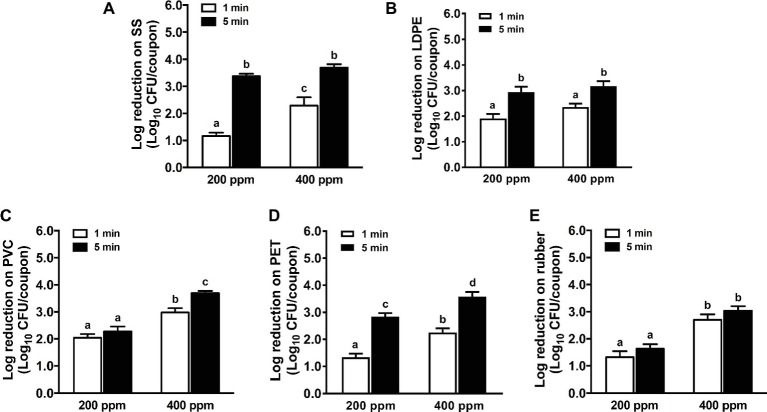
Antimicrobial efficacy of quaternary ammonium compound (QAC) against *L. monocytogenes* biofilms on food-contact surfaces. **(A)** Stainless steel (SS); **(B)** low-density polyethylene (LDPE); **(C)** polyvinyl chloride (PVC); **(D)** polyester (PET); **(E)** rubber. The 7-day-old biofilms on different surface coupons (15 mm × 7.5 mm) were treated with 200 or 400 ppm QAC for 1 or 5 min at 22°C. The surviving bacteria were shown as means ± SEMs, *n* = 3. ^a–d^Bars topped with the different letters are significantly different at *p* ≤ 0.05.

In general, increasing the QAC concentration from 200 to 400 ppm improved its efficacy against *L. monocytogenes* biofilms on different food-contact surfaces except LDPE surface for both 1- and 5-min exposures (Figure 1). A 5-min exposure of QAC at 200 or 400 ppm showed a similar efficacy against *L. monocytogenes* biofilms on SS coupons (Figure 1A). Except for rubber surface, the efficacy of QAC against *L. monocytogenes* biofilms on different surfaces was enhanced when exposure time increased from 1 to 5 min (Figure 1). Among all surfaces, QAC at 5 min exposure was the most effective against *L. monocytogenes* biofilms on SS (Figure 1A), least effective against *L. monocytogenes* biofilms on rubber (Figure 1E), while exhibiting a comparable efficacy against *L. monocytogenes* biofilms on LDPE and PET (Figures 1B–D). For *L. monocytogenes* biofilms on PVC surface, the 5-min exposure of 400 ppm QAC showed a similar efficacy as those of LDPE and PET; however, 200 ppm QAC for 5 min of exposure was less effective on PVC surface than those of LDPE and PET (Figures 1B–D). QAC at the FDA-approved concentration of 400 ppm for 5 min caused 3.7, 3.2, 3.7, 3.6, and 3.0 log_10_ CFU/coupon reductions of *L. monocytogenes* biofilms on SS, LDPE, PVC, PET, and rubber surface, respectively (Figure 1).

### Efficacies of Chlorine and Chlorine Dioxide Against *L. monocytogenes* Biofilms on Food-Contact Surfaces

**Figure 2:**
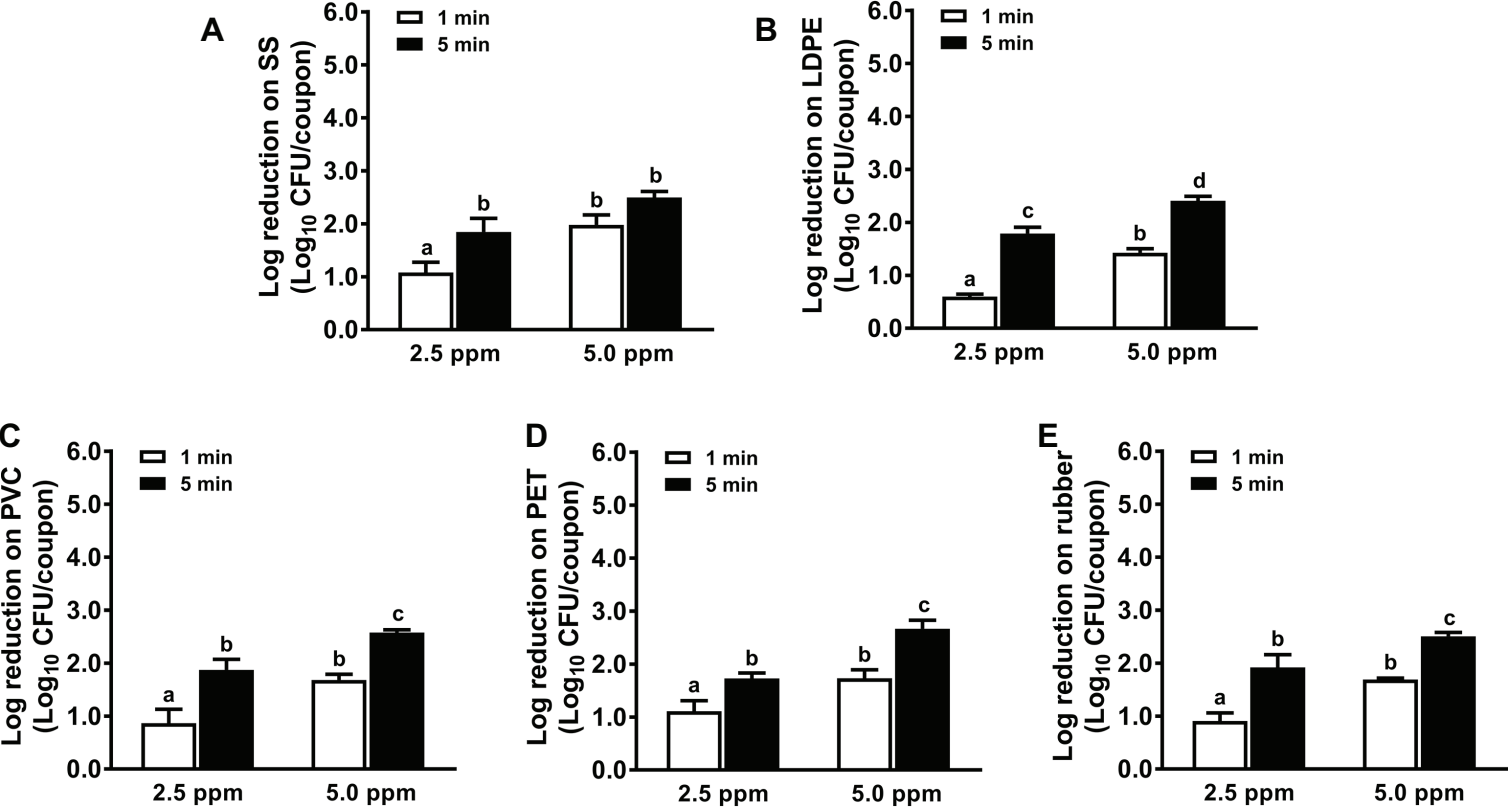
Antimicrobial efficacy of chlorine dioxide against *L. monocytogenes* biofilms on food-contact surfaces. **(A)** Stainless steel (SS); **(B)** low-density polyethylene (LDPE); **(C)** polyvinyl chloride (PVC); **(D)** polyester (PET); **(E)** rubber. The 7-day-old biofilms on different surface coupons (15 mm × 7.5 mm) were treated with 2.5 or 5.0 ppm chlorine dioxide solution for 1 or 5 min at 22°C. The remaining bacteria post-sanitizer treatment were shown as means ± SEMs, *n* = 3. ^a–d^Bars topped with the different letters are significantly different at *p* ≤ 0.05.

Chlorine dioxide solution at 2.5 ppm exhibited a limited efficacy against *L. monocytogenes* biofilms on all surfaces tested; 1-min treatments only reduced ~1.1, 0.6, 0.9, 1.1, and 0.9 log_10_ CFU/coupon *L. monocytogenes* biofilms on SS, LDPE, PVC, PET, and rubber surfaces, respectively (Figure 2). Though the efficacy of chlorine dioxide was enhanced with increased concentration and contact time, it displayed limited potency to inactivate *L. monocytogenes* biofilms on food-contact surfaces. A 5-min treatment of 5.0 ppm chlorine dioxide caused similar bactericidal efficacy against *L. monocytogenes* biofilms on all surfaces with 2.4–2.7 log_10_ CFU/coupon reductions (Figure 2).

**Figure 3:**
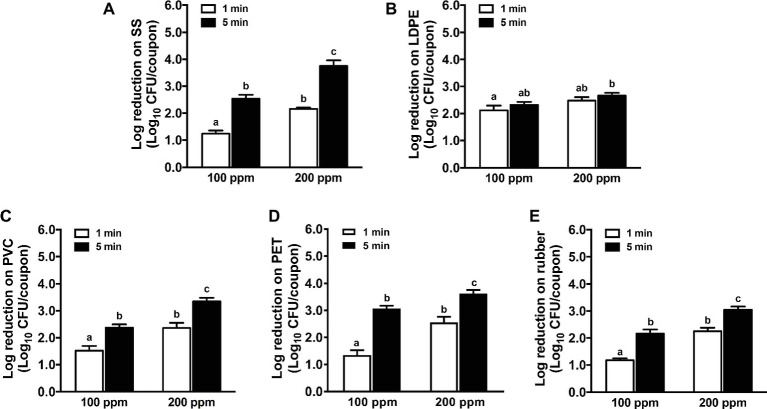
Antimicrobial efficacy of chlorine against *L. monocytogenes* biofilms on food-contact surfaces. **(A)** Stainless steel (SS); **(B)** low-density polyethylene (LDPE); **(C)** polyvinyl chloride (PVC); **(D)** polyester (PET); **(E)** rubber. The 7-day-old biofilms on different surface coupons (15 mm × 7.5 mm) were treated with 100 or 200 ppm chlorine solution for 1 or 5 min at 22°C. The survivors post-chlorine treatment were enumerated and shown as means ± SEMs, *n* = 3. ^a–c^Bars topped with the different letters are significantly different at *p* ≤ 0.05.

The efficacy of chlorine against *L. monocytogenes* biofilms on the tested surfaces was enhanced at increased concentration and extended contact time except LDPE surface (Figure 3). A 1-min treatment of 100 ppm chlorine showed a similar efficacy against *L. monocytogenes* biofilms as 1-min exposure of 200 ppm QAC (Figure 1) and was more effective than 1-min treatment of 2.5 ppm chlorine dioxide (Figure 2), causing 1.0–2.0 log_10_ CFU/coupon reductions of biofilms on all surfaces tested. Chlorine at 200 ppm for 5.0-min exposure caused 3.8, 2.7, 3.3, 3.6, and 3.0 log_10_ CFU/coupon reductions of *L. monocytogenes* biofilms on SS, LDPE, PVC, PET, and rubber surfaces, respectively (Figure 3).

### Efficacy of Peroxyacetic Acid Against *L. monocytogenes* Biofilms on Food-Contact Surfaces

**Figure 4:**
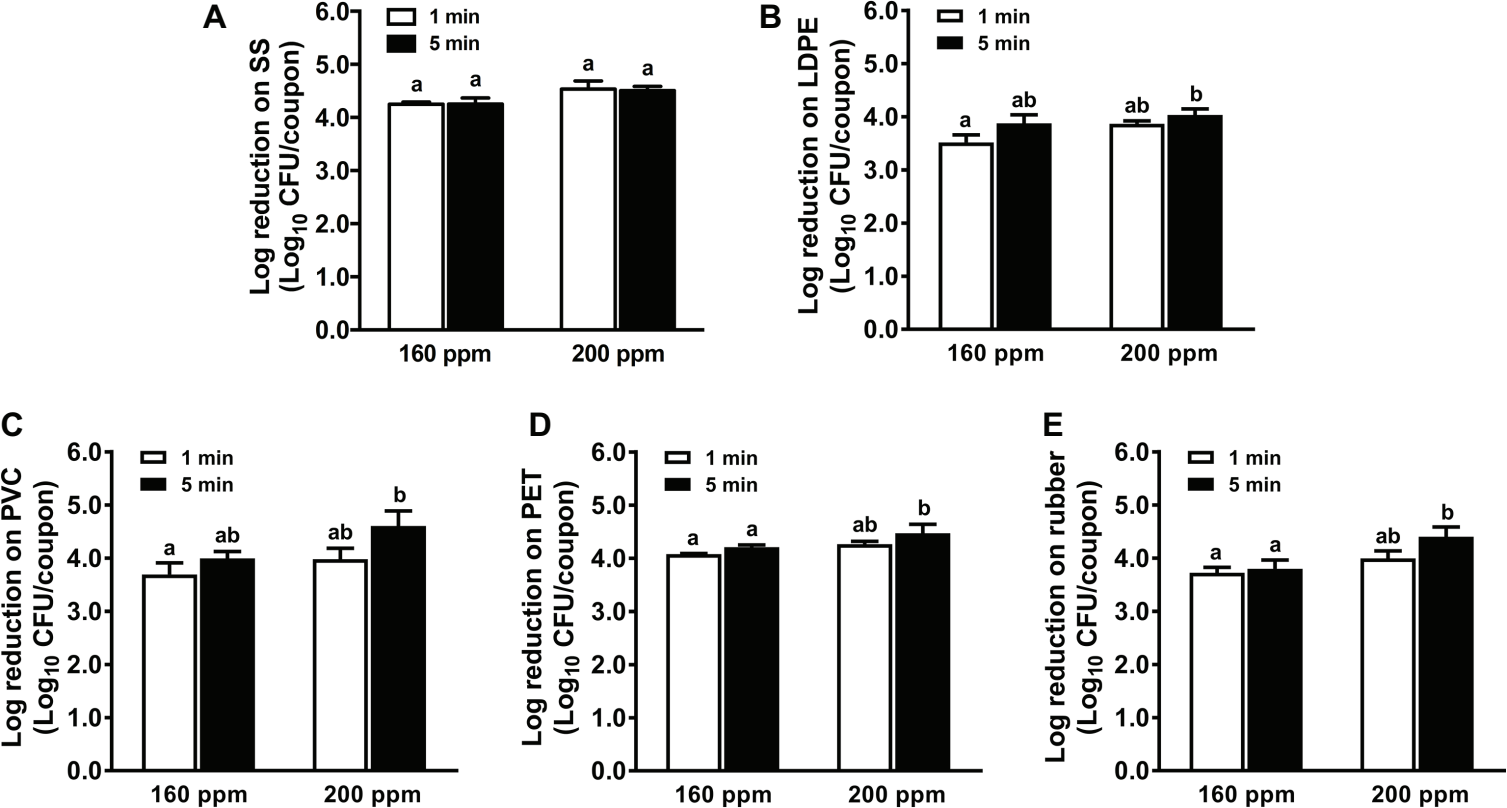
Antimicrobial efficacy of peroxyacetic acid (PAA) against *L. monocytogenes* biofilms on food-contact surfaces. **(A)** Stainless steel (SS); **(B)** low-density polyethylene (LDPE); **(C)** polyvinyl chloride (PVC); **(D)** polyester (PET); **(E)** rubber. The 7-day-old biofilms on different surface coupons (15 mm × 7.5 mm) were treated with 160 or 200 ppm PAA for 1 or 5 min at 22°C. The surviving bacteria were shown as means ± SEMs, *n* = 3. ^a,b^Bars topped with the different letters are significantly different at *p* ≤ 0.05.

Among all selected sanitizers, PAA was the most effective against *L. monocytogenes* biofilms on all food-contact surfaces (Figure 4). One min treatment of 160 ppm PAA reduced ~4.3, 3.5, 3.8, 4.1, and 3.7 log_10_ CFU/coupon *L. monocytogenes* biofilms on SS, LDPE, PVC, PET, and rubber surfaces, respectively (Figure 4). In general, the bactericidal effects of PAA against *L. monocytogenes* biofilms on all surfaces was not improved when the PAA concentration increased from 160 to 200 ppm or when the treatment time increased from 1 to 5 min (Figure 4). The 5-min treatment of 200 ppm PAA caused 4.5, 4.0, 4.4, 4.3, and 4.4 log_10_ CFU/coupon reductions of *L. monocytogenes* biofilms on SS, PET, PVC, LDPE, and rubber, respectively (Figure 4).

### Effects of Organic Matter on Sanitizer’s Efficacy

**Figure 5:**
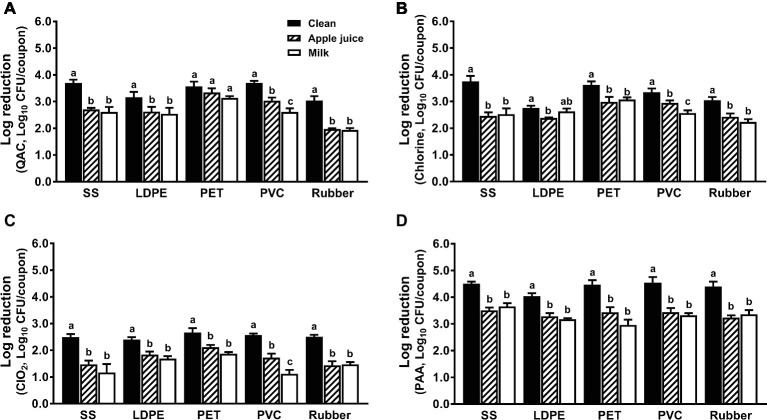
Efficacy of four commonly used sanitizers against *L. monocytogenes* biofilms on food-contact surfaces conditioned with organic matters. **(A)** Quaternary ammonium compound (QAC, 400 ppm); **(B)** chlorine (200 ppm); **(C)** chlorine dioxide (ClO_2_, 5.0 ppm); **(D)** peroxyacetic acid (PAA, 200 ppm); stainless steel (SS); low-density polyethylene (LDPE); polyester (PET); polyvinyl chloride (PVC). Apple juice: food-contact surfaces were conditioned with apple juice; milk: food-contact surfaces were conditioned with milk. The 7-day-old biofilms on different surface coupons (15 mm × 7.5 mm) were treated with the respective sanitizers for 5 min, then survivors were enumerated and shown as means ± SEMs, *n* = 3. ^a–c^Bars topped with the different letters are not significantly different at *p* ≤ 0.05.

The anti-*Listeria* efficacies of all sanitizers were compromised by organic matter regardless of surfaces tested; food residues from apple juice or milk comparably impacted QAC efficacy (Figure 5). Soiling has a greater influence on the antimicrobial efficacy of QAC against biofilms on SS and rubber than those on LDPE, PET, and PVC (Figure 5A). Among all tested surfaces, the anti-*Listeria* efficacy of chlorine on SS is the most impacted by organic matter. Chlorine at 200 ppm and 5-min contact time showed a similar anti-*Listeria* efficacy on soiled SS, LDPE and rubber surfaces regardless of organic matter type (Figure 5B). The bactericidal effect of chlorine dioxide against *L. monocytogenes* biofilms was compromised by organic matter regardless of surface materials or food residue source. Chlorine dioxide at 5.0 ppm for 5 min caused 1.0–2.0 log_10_ CFU/coupon reduction depending on surface material (Figure 5C). Though the PAA efficacy against *L. monocytogenes* biofilms on all surfaces was impaired by organic soiling as much as other sanitizers, it was still the most effective sanitizer, which caused 3.0–3.7 log_10_ CFU/coupon reductions of *L. monocytogenes* biofilms on different surfaces (Figure 5D).

## Discussion

### The Effect of Concentration, Contacting Time of Sanitizers on Inactivation of *L. monocytogenes*

The concentrations of QAC, chlorine dioxide, chlorine and PAA against *L. monocytogenes* biofilms on common food-contact surfaces were selected complying with FDA regulation (FDA, 2017). The 200 ppm QAC, 2.5 ppm chlorine dioxide, or 100 ppm chlorine interventions showed limited efficacies against aged *L. monocytogenes* biofilms on different food-contact surfaces, but their efficacies were enhanced with increased concentrations, which was consistent with our previous findings on polystyrene surface (Korany et al., 2018) and other studies on SS surface (Robbins et al., 2005; Trinetta et al., 2012; Dhowlaghar et al., 2018). The antimicrobial efficacies of QAC, chlorine dioxide, and chlorine at selected concentrations were improved when increasing contact time from 1 to 5 min, which is supported by a recent report of QAC and chlorine against *L. monocytogenes* biofilms on SS surface (Dhowlaghar et al., 2018). Similarly, the efficacy of chlorine dioxide in aqueous and gaseous phase against *L. monocytogenes* biofilms on food contact surfaces increased with extended contact time (Vaid et al., 2010; Trinetta et al., 2012; Park and Kang, 2017). Increasing PAA concentration from 160 to 200 ppm or extending the contacting time from 1 to 5 min at selected concentration did not improve its efficacy in general. A similar result was obtained for *L. monocytogenes* biofilms on polystyrene surfaces (Korany et al., 2018). Compared with QAC, chlorine, and chlorine dioxide, PAA tested in the present study was the most effective sanitizer against aged *L. monocytogenes* biofilms on all surfaces, which was consistent with findings on polystyrene (Korany et al., 2018), SS (Dhowlaghar et al., 2018), and PVC (Berrang et al., 2008). It could be due to its high reactivity, oxidizing capacity, decomposition rate, and low molecular weight, which together allow PAA to penetrate biofilm matrix, thus accomplishing bactericidal activity (Ibusquiza et al., 2011).

### Effects of Surface Materials on Efficacy of Different Sanitizers Against *L. monocytogenes*

The efficacies of sanitizers against aged *L. monocytogenes* biofilms varied on different surfaces. The 1-min treatment of QAC or chlorine at selected concentrations caused comparative efficacies against *L. monocytogenes* biofilms on SS, PET, and rubber, which is supported by a previous report on polystyrene surface (Korany et al., 2018). Compared with rubber and LDPE, 400 ppm QAC and 200 ppm chlorine at 5-min exposure were more effective against *L. monocytogenes* biofilms on SS and other surfaces. In support of our finding, *L. monocytogenes* on rubber surface was more difficult to remove by chlorine, QAC, and chlorine dioxide than that on SS surface (Ronner and Wong, 1993; Park and Kang, 2017). Different from QAC and chlorine, the anti-*Listeria* effects of PAA and chlorine dioxide were minimally influenced by surface material at different concentration and time combinations. Regardless of surfaces, chlorine dioxide at 5.0 ppm showed a 2.5 log reduction after 5-min treatment, which is a very limited efficacy in contrast to 4.0 or more reduction caused by 200 ppm PAA at 5-min contact. Similar to our results, the aerosolized PAA exhibited similar antimicrobial efficacy against *L. monocytogenes* biofilms on SS and PVC surfaces, though the efficacy was lower than our finding (Park et al., 2012). Each type of surface material has different topography and roughness that provide unique microcracks/harbor sites for *L. monocytogenes* and protect the entrapped cells from antimicrobial agents (Chaturongkasumrit et al., 2011; Schlisselberg and Yaron, 2013), which might explain the difference in efficacy against biofilms on different surfaces. In support, 20 ppm gaseous chlorine dioxide was more effective against attached *L. monocytogenes* on glossy SS than coarse SS, and *Salmonella* biofilms on smooth SS were more susceptible to 50 ppm chlorine treatment than those on a rough surface (Schlisselberg and Yaron, 2013). Surface materials with different hydrophobicity and hydration levels lead to various sanitizing efficacy; hydrophobic surface was more difficult to clean than hydrophilic surface (Park and Kang, 2017).

### The Antimicrobial Efficacy of Sanitizers in the Presence of Organic Matter

Food residues established on food-contact surfaces alter the physicochemical property of these surfaces and impact sanitizer efficacy (Abban et al., 2012; Brown et al., 2014). The present study indicated that organic soiling, regardless of sources, impaired efficacies of all sanitizers against biofilms on all food-contact surfaces, which is consistent with the finding on polystyrene surface (Korany et al., 2018). In agreement with our findings, protein and fat residues on SS reduced the efficacies of chlorine dioxide (Vandekinderen et al., 2009), hydrogen peroxide (Moretro et al., 2019), acidic electrolyzed water and sodium hypochlorite (Ayebah et al., 2006), QAC, chlorine, and PAA (Aarnisalo et al., 2000; Somers and Wong, 2004; Kuda et al., 2008) against *L. monocytogenes* biofilms. Besides attracting bacterial cells as an adhesive layer, protein coating reduced water contact angle, leading to decreased hydrophobicity of food-contact surface (Abban et al., 2012; Park and Kang, 2017). In addition, sanitizers may have difficulty reaching bacterial cells due to the physical and chemical barriers built up by exopolysaccharide substance of biofilm matrix together with food residues (Fernandes et al., 2015).

## Conclusion

The type of surface material has more dramatic effects on anti-*Listeria* efficacy of QAC and chlorine than those treated with chlorine dioxide and PAA. Food residue soiling, regardless of sources, reduced anti-*Listeria* efficacies of all sanitizers against biofilms on surfaces in general. Among all sanitizers, PAA was the most effective sanitizer against *L. monocytogenes* biofilms on different surfaces. A 5-min treatment of 200 ppm PAA resulted in 3.0–3.7 log_10_ reductions of aged multi-strain *L. monocytogenes* biofilms on major food contact surfaces in the presence of organic matter. Data once again highlight the importance of thorough cleaning of food-contact surfaces prior to sanitizer interventions and provide useful information for food industries in selecting appropriate sanitizers for food-contact surfaces’ decontamination.

## Data Availability Statement

The datasets generated for this study are available on request to the corresponding author.

## Author Contributions

ZH and AK conducted the experiments. ZH wrote the manuscript. M-JZ designed the study. M-JZ and SE-S revised the manuscript.

### Conflict of Interest

The authors declare that the research was conducted in the absence of any commercial or financial relationships that could be construed as a potential conflict of interest.
